# Using the Multi-Theory Model (MTM) of Health Behavior Change to Explain the Seeking of Stool-Based Tests for Colorectal Cancer Screening

**DOI:** 10.3390/ijerph20166553

**Published:** 2023-08-10

**Authors:** Manoj Sharma, Christopher Johansen, Kavita Batra, Chia-Liang Dai, Ravi Batra, Traci Hayes, Aditi Singh

**Affiliations:** 1 Department of Social and Behavioral Health, School of Public Health, University of Nevada, Las Vegas, NV 89119, USA; manoj.sharma@unlv.edu; 2 Department of Internal Medicine, Kirk Kerkorian School of Medicine, University of Nevada, Las Vegas, NV 89102, USA; aditi.singh@unlv.edu; 3 Office of Research, Kirk Kerkorian School of Medicine, University of Nevada, Las Vegas, NV 89102, USA; 4 Department of Medical Education, Kirk Kerkorian School of Medicine, University of Nevada, Las Vegas, NV 89102, USA; 5 Department of Teaching and Learning, College of Education, University of Nevada, Las Vegas, NV 89102, USA; chia-liang.dai@unlv.edu; 6 Department of Environmental and Occupational Health, School of Public Health, University of Nevada, Las Vegas, NV 89119, USA; batrar2@unlv.nevada.edu; 7 Department of Public Health, School of Health Professions, University of Southern Mississippi, Hattiesburg, MS 39406, USA; traci.hayes@usm.edu

**Keywords:** multi-theory model, screening, colorectal cancer, polyps

## Abstract

Colorectal cancer is the third most common cancer worldwide and is the second leading cause of cancer-associated deaths. While colorectal cancer is on the decline in the United States (US), disparities still exist, despite the non-invasive screening modalities, such as stool-based tests have shown themselves to be effective in the detection of colorectal cancer. Many of the existing stool-based test interventions lack the use of a contemporary theory-based approach. Given the paucity of theory-based interventions intended to promote stool-based tests, this cross-sectional study utilizes the multi-theory model (MTM) of health behavior change to explain the seeking of stool-based tests for colorectal cancer (CRC) screening. An online 57-item questionnaire with an established psychometric validity was used to collect responses from the US-based sample (*n* = 640) of adults aged 45–75 years old. The data were analyzed using bivariate and multivariate statistical methods. Structural equation modeling (SEM) was conducted to test the construct validity of the survey instrument. In this nationwide sample, 39.2% (*n* = 251) of participants reported having received some form of a stool-based test. Among the participants who did not undergo stool-based CRC screening, the MTM subscales, including “participatory dialogue”, “behavioral confidence”, and “changes in the social environment”, were significant predictors of initiating screening behavior and explained 48% of the variance in the initiation among this group (R^2^ = 0.579, F = 5.916, *p* < 0.001; adjusted R^2^ = 0.481). The MTM may be a useful framework with which to design educational, mass media, social media, and clinical interventions for the promotion of stool-based CRC screening among adults aged 45–75 years old.

## 1. Introduction

Colorectal cancer (CRC) is among the most common cancers and sources of cancer-associated mortality worldwide [1]. Globally, an estimated 1.9 million cases of CRC were diagnosed, leading to 900,000 deaths in 2020 [1]. In 2023, approximately 153,000 individuals will be diagnosed with CRC, and 52,550 will die from CRC [2]. CRC is on the decline in the United States (US); however, the incidence from 2015 to 2019 was 33% higher for men than women [2]. Further, the incidence and mortality rates are highest for certain racial/ethnic minority groups, such as American Indian/Alaskan Natives, and African Americans [2]. On the other hand, Asian American/Pacific Islanders had the lowest CRC incidence and mortality [2]. Despite advances in screening, individuals in the US are being diagnosed with CRC at a younger age and at later stages [2]. Mortality is improved with early diagnosis and treatment of CRC [1,2,3].

Stool-based screening modalities have been shown to be effective, less invasive tests in the detection of CRC rates; they include the fecal immunochemical test (FIT), guaiac-based occult blood test (gFOBT), and multitarget stool DNA (mt-sDNA) [1,2,3]. The US Preventive Services Task Force 2021 Guidelines recommends stool-based screenings to be conducted annually for gFOBT and FIT or every three years for the mt-sDNA [1,4].

Stool-based interventions have been utilized to detect CRC, and many have examined the uptake of FIT, gFOBT, and mt-sDNA [5,6,7,8,9,10,11,12,13,14,15]. For example, prior studies found that outreach reminders/invitations via mail were associated with greater FIT screenings as compared to usual care [6,8]. Other interventions focused on patient navigation [8,12,13] and patient education [16,17]. Some studies found that fecal blood test outreach and patient navigation, particularly in the context of multicomponent interventions, were associated with increased CRC screening rates in US based trials [7]. However, many of these studies lacked the use of a contemporary theory-based approach. In a systematic review and meta-analysis of 73 interventions, there were few mentions of theoretical frameworks or approaches used [7]. A few studies included theoretical approaches relied on the health belief model (HBM), transtheoretical model (TTM), theory of planned behavior (TPB), and general model of the determinants of behavioral change [11]. There is a need to utilize theory in CRC intervention research due to the limited interventions that used theoretical paradigms to explain CRC screening behaviors among US adults.

It is vital to select an appropriate theory in CRC research. Therefore, it is possible that the fourth-generation multi-theory model (MTM) of health behavior change may explain different modalities of CRC screening [18]. The MTM postulates that behavior change consists of (1) initiation and (2) sustenance or maintenance [18,19,20]. In initiation, an individual must be convinced that the advantages outweigh the disadvantages (*participatory dialogue*), must have behavioral confidence, and must have support from the physical environment. In the maintenance of behavior change, an individual must be able to transform his or her emotions into goals, continually strive for change (*practice for change*), and must have support from their social environment. The theory has been used in a variety of health behaviors, including mammography screening, HPV vaccination, and cervical cancer screening [19,20,21]. For this study, we focused on explaining the seeking of stool-based colorectal cancer screening tests (FIT, gFOBT, and mt-sDNA) among people between the ages of 45 and 75 years using a modified version of MTM in which the initiation model was bolstered with the construct of *changes in the social environment* from the sustenance model due to the sporadic nature of the behavior. This was the first study using MTM with CRC screening among US adults aged 45–75 years.

## 2. Materials and Methods

### 2.1. Study Design and Participants’ Recruitment

**Figure 1:**
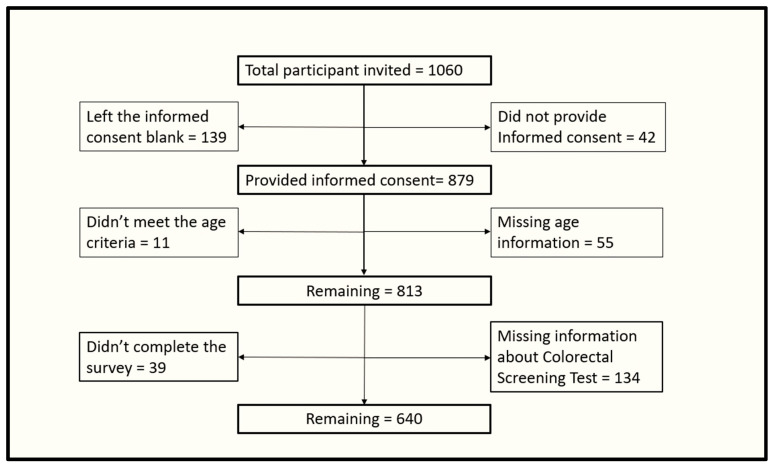
Flowchart detailing the sample recruitment.

This was a cross-sectional study which utilized a sample of US adults aged 45–75 years who understand English language. The sample selection process has been described in Figure 1. The study samples were obtained from currently available pools of research participants, who have consented to be contacted for future studies. To avoid heavy dependence on a single segment of the population, Qualtrics pooled samples from different sources across the nation. Recruitment of respondents was conducted in an enforced quota sampling fashion to create a pool of participants representing the current US population demography.

### 2.2. Sampling and Data Collection

Data for this study were collected from January to March 2023 via a web-based structured survey built in Qualtrics hosting platform (Provo, UT, USA). The Qualtrics Research Marketing team collected the data as a part of the contractual agreement set up between researchers and the Qualtrics team. The survey link was distributed through multiple avenues, including in-app notifications, listserv, among the contacts of the investigators, etc.

### 2.3. Ethical Considerations

The Institutional Ethics Committee of the University of Nevada, Las Vegas, granted the exempt status (protocol ID: UNLV-2022-575) on 23 December 2022. All ethical guidelines in accordance with the Declaration of Helsinki were followed. There were no personal identifiers collected to ensure the anonymity of the participants. Participants were provided with detailed information about the purpose, objectives, procedure, benefits, and risks associated with this study, which could help them make informed decisions to participate in the study. Participation in this study was completely voluntary and participants could drop out any time as they wish. To prevent multiple responses from the same participant (duplicate responses), strategies such as digital fingerprinting and the “prevent multiple responses option in Qualtrics” were used. Screening questions that were aligned with the inclusion criteria were posed at the beginning of the survey to avoid any self-selection bias. For participants who completed the survey, incentives in the form of gift cards, cash rewards, SkyMiles, or redeemable points were given per the contract between Qualtrics and panel providers.

### 2.4. Questionaire

**Figure 2:**
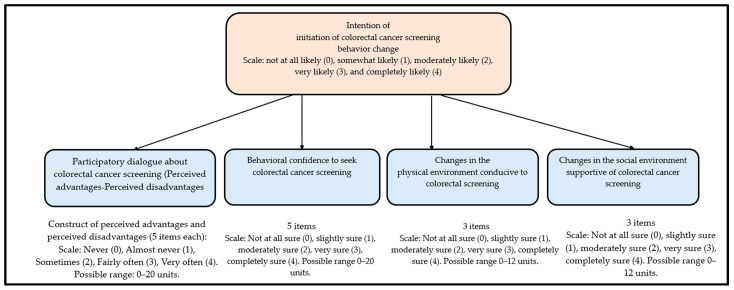
MTM framework for the colorectal cancer screening behavior change.

This 57-item survey tool (see Supplementary Materials) used in this study was grounded in the MTM framework [18,22]. Originally, there are two main components of the MTM, namely, “initiation”, and “sustenance”. However, for this study, only the “initiation” component was conceptualized. In this tool, “initiation” has four constructs, namely, “participatory dialogue”, “behavioral confidence”, “changes in the physical environment”, and “changes in the social environment”. Please see Figure 2 for a detailed overview of the MTM framework. The first construct of the “participatory dialogue” is derived from the “perceived advantages”, and “perceived disadvantages” (mathematically: “participatory dialogue” score = “perceived advantages” score minus (−) “perceived disadvantages” score). There were 5 items each for the “perceived advantages” and “perceived disadvantages”, which were measured on a 5-point Likert scale with a possible range of 0–20 units. Another construct was the “behavioral confidence” that was measured on the surety scale with a possible range of 0–20 units too. This construct measures confidence to initiate a certain behavior. The third construct was the “changes in the physical environment”, which points to the tangible resources required to initiate a certain kind of behavior. This was measured through 3 items on a 5-point Likert scale. The last construct of “changes in the social environment” was based on the premise of social influence on initiating a behavior, which was measured on the 5-point Likert scale with 3 items.

### 2.5. Survey Validation

**Figure 3:**
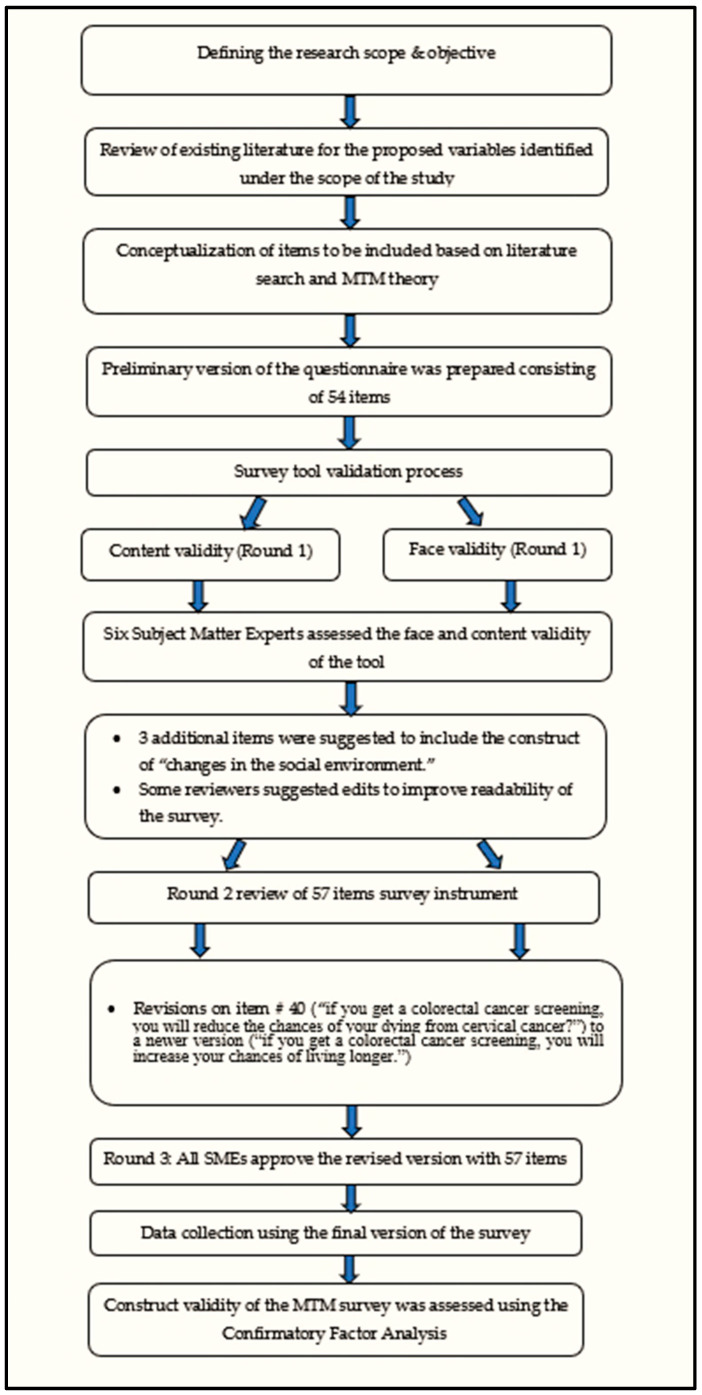
A flowchart detailing the steps of survey validation.

The survey underwent face and content validation by six subject matter experts (of which five were university faculty with multiple expert areas) in the fields of behavioral theories (*n* = 3), colorectal cancer (*n* = 2), target population experts (*n* = 2), and survey validation (*n* = 4). Since the experts had multiple expertise, the numbers listed in the parentheses above may not sum to a total of six. The initial version included a total of 54 items. Experts provided comments to establish the face and content validity of the instrument and improve the readability/clarity of the survey, and 3 additional items were included for the construct of “changes in the social environment” to the survey. The rest of the changes entailed some wordsmithing in order to improve the readability for the target population. There was consensus among experts after three rounds of reviews to finalize a 57-item instrument for the current study. All steps of survey validation can be seen in Figure 3.

### 2.6. Construct Validity

Structural equation modeling (SEM) was performed by using Mplus to examine the construct validation of the measurement model. The hypothesized model included advantages, disadvantages, behavioral confidence, changes in the physical environment, and changes in the social environment as independent variables, and initiation of health behavior change as the dependent variable. The chi-square (*x*^2^), comparative fit index (CFI), root mean square error of approximation (RMSEA), and standardized root mean square residual (SRMR) indices were reported to indicate the goodness of fit [23]. We interpreted CFI and TLI > 0.90 [24,25] and RMSEA and SRMR < 0.08 [26] as evidence of acceptable model fit.

### 2.7. Testing of Assumptions

Assumptions of independence of observations (i.e., Durbin–Watson statistic), linearity (e.g., scatterplot and partial regression plots), homoscedasticity, multicollinearity, and normality (i.e., P–P plot and Q–Q plot) were evaluated in this study before selecting the model. Homogeneity of variance assumption was assessed by the Levene’s test. There was independence of residuals, as assessed via a Durbin–Watson statistic of nearly 2. There was homoscedasticity, as assessed via visual inspection of a plot of studentized residuals versus unstandardized predicted values. There was no evidence of multicollinearity, as assessed via tolerance values greater than 0.1. There were no studentized deleted residuals greater than ±3 standard deviations, no leverage values greater than 0.2, and values for Cook’s distance above 1. The assumption of normality was met, as assessed by a P–P Plot.

### 2.8. Data Analysis

Univariate, bivariate, and multivariate statistical tests were used to analyze the data. Frequencies/proportions were used to represent categorical variables, whereas continuous variables were represented as the means and standard deviations given the normal distribution. For initiation, two separate models of hierarchical multiple regression analyses were used among those participants who underwent stool-based colorectal screening and those who had not undergone these screening tests. The selection of variables to be entered in the regression was assessed based on a theoretical, conceptual, and statistical basis. All tests were performed using IBM SPSS (version 28.0), with the level of significance set at 5%.

In calculating the sample size, we used the following parameters: confidence level: 95; margin of error: 5%; and population proportion: 33%. The population proportion of (100 − 67) = 33% was based on the data reported by the Centers for Disease Control and Prevention in 2018 [27], according to which 67% of US adults aged 50–75 years met the recommendations of colorectal cancer screening. The minimal sample required was 340, and after applying a 20% non-response rate, a total of *n* = 340 + 68 = 408 was needed. Our sample size is sufficiently larger than estimated, which allowed us to conduct structural equation modeling too [28].

## 3. Results

### 3.1. Construct Validity

**Figure 4:**
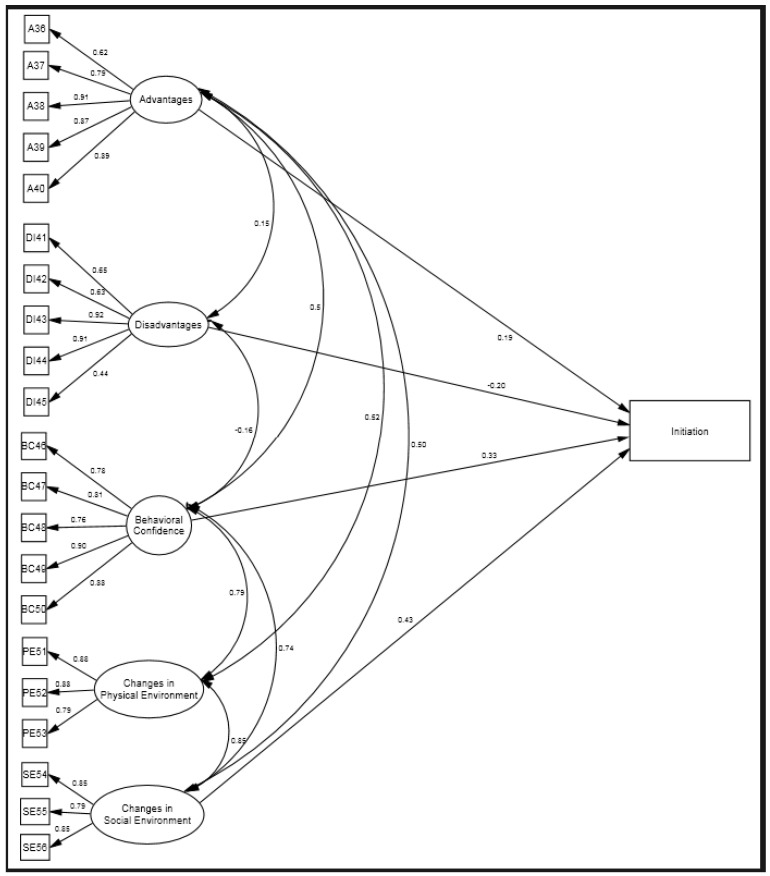
A structural model of initiating colorectal cancer screening behavior. Only statistically significant factor loadings were displayed. A: Advantages; DI: Disadvantages; BC: Behavioral Confidence; PE: Changes in Physical Environment; SE: Changes in Social Environment.

The fit indices suggested an acceptable fit of the initiation model (*x*^2^ [195] = 767.145 (*p* < 0.001), CFI = 0.923, TLI = 0.908, RMSEA = 0.07, and SRMR = 0.054). First, we examined the measurement model and observed an overall pattern of statistically significant factor loadings of five latent variables: advantages; disadvantages; behavioral confidence; changes in the physical environment; and changes in the social environment. Specifically, the variable of advantages had large effects (e.g., β ranging from 0.62 to 0.91) on its indicators; the variable of disadvantages had moderate-to-large effects (e.g., β ranging from 0.44 to 0.92) on its indicators; and the variable of behavioral confidence had large effects (e.g., β ranging from 0.76 to 0.90) on its indicators as well. The variables of changes in the physical environment and changes in the social environment each had large effects on their respective indicators, with βs above 0.79 in Figure 4. These effects indicated that the scale provided a valid measurement of intended constructs. Second, we examined the construct correlations and standardized regression coefficients and found that advantages, behavioral confidence, and changes in the social environment were positively associated with (e.g., β ranging from 0.19 to 0.43, *p* < 0.001), and disadvantages were negatively associated with (β = −0.20, *p* < 0.001) the initiation of colorectal cancer screening behavior. However, the effects of changes in the physical environment on initiation were statistically insignificant.

### 3.2. Characteristics of the Sample

In a total sample of 640 participants, 251 (Group 1: 39.2%) reported having any sort of stool-based colorectal cancer screening tests, while 389 (Group 2: 60.8%) reported not having any sort of stool-based colorectal cancer screening tests (Table 1). Nearly 50% of the sample constituted Whites and Christians and were married. The sample consisted predominately of females (57.6%), non-Hispanics (80.2%), and those with health insurance (88.5%) (Table 1). Participants who have had any form of stool-based colorectal screening tests (group 1) had a significantly lower proportion of uninsured individuals than those who have not had any form of stool-based colorectal screening tests (group 2) (2.7% vs. 8.1%, *p* = 0.009, Table 1). A significantly higher proportion of those who underwent any sort of stool-based colorectal cancer screening had a personal history of colorectal cancer (5.6% vs. 1.1%, *p* = 0.001), had a personal history of inflammatory bowel diseases (13.0% vs. 6.2%, *p* = 0.005), had a personal history of confirmed or suspected hereditary colorectal cancer syndrome (7.0% vs. 1.7%, *p* = 0.001), had a history of abdominal irradiation (9.2% vs. 2.6%, *p* < 0.001), and were recommended a colorectal screening by their healthcare providers (45.5% vs. 28.7%, *p* < 0.001, Table 2).

### 3.3. Comparison of the MTM Constructs

As shown in Table 3, the mean scores of initiation—“perceived advantages”, (14.19 ± 5.09 vs. 13.04 ± 5.47, *p* = 0.01), “behavioral confidence”, (12.10 ± 5.34 vs. 11.11 ± 5.53, *p* = 0.03), “changes in the physical environment”, (8.45 ± 3.31 vs. 7.64 ± 3.70, *p* = 0.006), and “changes in the social environment”, (7.51 ± 3.61 vs. 6.60 ± 3.87, *p* = 0.004)—were statistically significantly higher in group 1 than in group 2.

### 3.4. Correlation and Reliability Diagnostics

As shown in the Table 4, “participatory dialogue” was positively and moderately correlated with the “behavioral confidence” (R^2^ = 0.51, *p* < 0.001), “changes in physical environment” (R^2^ = 0.45, *p* < 0.001), and “changes in the social environment” (R^2^ = 0.42, *p* < 0.001). Behavioral confidence had a strong positive correlation with the “changes in physical environment” (R^2^ = 0.75, *p* < 0.001) and “changes in social environment” (R^2^ = 0.68, *p* < 0.001). The “change in physical environment” was strongly and directly correlated with the “changes in the social environment” (R^2^ = 0.77, *p* < 0.001, Table 4). The overall reliability (Global Cronbach’s Alpha) of the tool was 0.942, and for the subscales, it ranged from 0.87 to 0.91, which indicates excellent reliability.

### 3.5. Hierarchical Regression

Among the participants who underwent stool-based CRC screening, the MTM subscales, including “participatory dialogue”, “behavioral confidence”, and “changes in the social environment”, were significant predictors of initiating screening behavior and explained 61% of the variance in the initiation among this group (R^2^ = 0.625, F = 15.730, *p* < 0.001; adjusted R^2^ = 0.609) (model 4, Table 5). Another significant predictor was the urban residence that had a conditional mean of (0.601 (intercept) + 0.358 (slope) = 0.959)). In other words, an average participant living in an urban area has a 0.358 points higher score of initiation than those living in rural areas.

Among the participants who did not undergo stool-based CRC screening, the MTM subscales, including “participatory dialogue”, “behavioral confidence”, and “changes in the social environment”, were significant predictors of initiating-screening behavior and explained 48% of the variance in the initiation among this group (R^2^ = 0.579, F = 5.916, *p* < 0.001; adjusted R^2^ = 0.481) (model 4, Table 6). Another significant predictor was the AAPI race, which had a conditional mean of (1.171 (intercept) + 0.726 (slope) = 1.897)). In other words, on average, the AAPI race has a 0.726 points higher score of initiation than the Black race.

## 4. Discussion

The study aimed to explain the correlates of seeking stool-based screening tests for colorectal cancer among adults aged 45–75 years using the modified initiation theoretical paradigm of the multi-theory model (MTM) of health behavior change. The interpretation of the results is divided into two groups: (1) those who had stool-based CRC screening tests; (2) those who did not have stool-based CRC screening tests. Both of these interpretations are complementary to each other.

Since MTM is about health behavior change, we will first interpret the results for those who did not have any form of stool-based CRC screening tests. It is worth noting that the constructs of *participatory dialogue*, *behavioral confidence*, and *changes in the social environment*, along with being of Asian American and Pacific Islander (AAPI) racial denomination, were positive and statistically significant explanatory variables; these accounted for nearly 48% of the variance in the intent to seek stool-based CRC screening tests. This amount of explanation is considered to be fairly high in behavioral and social sciences [22]. It is evident from these findings that being convinced of advantages (such as early detection of CRC, increased chances of cure, having peace of mind, reduction in worries, chances of living longer, and so on) over disadvantages (*participatory dialogue*) is crucial in swaying the decisional balance for those who have not yet taken stool-based CRC screening tests. Similarly, the ability to proactively seek these CRC screening tests at prescribed intervals with conviction despite difficulties (*behavioral confidence*) is also vital in motivating individuals who have not yet taken the stool-based CRC screening tests to take the tests. Likewise, support from family, friends, healthcare professionals, social media, and so on (*changes in the social environment*) is also essential for motivating people to take stool-based CRC screening tests. These findings are in consonance with previous work based on other theories such as HBM, TTM, and TPB [11]. In demographic variables, our findings indicated that AAPI were more inclined to take stool-based CRC screening tests than Blacks. AAPIs have the lowest incidence and mortality due to CRC among all races [2]. Perhaps the lower mortality could be explained due to the early detection of polyps in this racial group. Health insurance status was also a predictor of obtaining a stool-based CRC screening test which aligns with previous findings [29]. Increased efforts to ensure equitable access to health are needed. Our study did not find *changes in the physical environment* or any other demographic variables to be significant predictors of the intent to seek stool-based CRC screening among adults aged 45–75 years who had not yet taken these tests in the final model.

As mentioned earlier, the findings from those who had stool-based CRC screening in the past were found to be complementary to the findings explained earlier. Once again, the MTM constructs of *participatory dialogue*, *behavioral confidence*, and *changes in the social environment* were statistically significant and accounted for almost 61% of the variance in the intent to seek stool-based CRC screening when due. As pointed out earlier, previous evidence from work in this area using HBM, TTM, and TPB is supportive of our findings [11]. The only difference in the findings among those who had CRC screening tests in the past that was noted with regard to demographic variables was that race was not significant. However, the predicted value for urban areas was found to be higher than for rural areas in the final model. This can perhaps be explained due to higher awareness of CRC and its screening in urban areas compared to rural areas.

Findings from the participants’ mean MTM scores revealed that neither group reached the highest possible scores, suggesting room for improvement. This can be conducted through educational interventions which can be used to increase scores in the MTM constructs. Further, all of the MTM construct’s mean scores were higher in directionality for those who had any form of stool-based CRC screening compared to those who did not. These results also support the inferential findings; these present a credible case for designing interventions using MTM to promote stool-based CRC screening tests. The findings for pre-existing conditions and their association with stool-based screening tests are also worth noting. The proportion of individuals with a personal history of CRC, a history of IBD, a history of hereditary CRC syndrome, a history of radiation to the abdomen, recommendations from healthcare providers, and a recent visit to a healthcare provider were all significantly higher among those who had a history of stool-based CRC screening compared to those who did not. These findings point to increased awareness and motivation among those who have personal adverse experiences or have been motivated by healthcare providers. This can be capitalized by future interventions to promote stool-based CRC screening tests among adults aged 45–75 years.

### 4.1. Implications for Practice

MTM seems to be a useful framework with which to design educational, mass media, social media, and clinical interventions promoting stool-based CRC screening among adults aged 45–75 years. For educational interventions, the advantages of stool-based CRC screening need to be facilitated through face-to-face settings or asynchronous/synchronous online settings. These advantages include highlighting early detection of CRC through these tests, increased chances of a cure if detected early, developing peace of mind, reduction in worries, chances of living longer, and so on. Moreover, in educational interventions, behavioral confidence can be built by demystifying the screening tests and identifying and remediating possible barriers to obtaining them. To influence changes in the social environment, family support, friends’ support, and healthcare professionals’ support should be actively encouraged.

In mass media and social media campaigns, messaging is very important. Succinct and clear messages based on MTM constructs can be crafted for dissemination. For example, a single message about an advantage of stool-based Cologuard can be, “*Convenience of home-based detection of colorectal cancer now within reach*! *Visit your primary care provider*”. This can be supplemented with a visual appeal.

Finally, interventions can be designed to be delivered by clinicians in clinical settings. Once again, these need to be succinct, brief, and to the point in order that the providers can easily incorporate these into their busy schedules. These can consist of a crib sheet consisting of 2–3 lines to be emphasized in communication with the patient that is readily accessible to the provider as a reminder to be read at each visit. Table 7 below summarizes a possible script for suggesting Cologuard to an eligible patient.

### 4.2. Strengths and Limitations of Our Study

Ours is the first study using the fourth-generation framework of MTM in the conceptualization and synthesis of data about the correlates of stool-based screening tests for CRC in adults aged 45–75 years. Our study was able to explain a substantial amount of the variance in the intent to take stool-based CRC screening tests in our target population. Our study was able to provide definitive implications for designing interventions in order to promote stool-based CRC screening tests in our target population. However, our study did have some limitations. First, we did not analyze the data pertaining to visual (structural) tests such as colonoscopy, virtual colonoscopy, or flexible sigmoidoscopy. Second, we used a cross-sectional study design, which gave us quick results but precludes us from making causal inferences due to a lack of established temporality. Third, we relied on self-reports that are amenable to several biases. However, our study was primarily based on identifying attitudes, and there could be no other means by which to do so. Next, as part of the contractual agreement with Qualtrics, partial responses were not provided, which limited our ability to analyze the characteristics of the non-responders, which is important for understanding the sources of non-response bias. Finally, while we established the face content and construct validity of our instrument, along with the internal consistency reliability, we could not establish test–retest reliability. This is something future researchers should attempt before embarking on intervention research.

## 5. Conclusions

This study reified the constructs of the modified initiation framework of the multi-theory model (MTM) of health behavior change in order to explain the correlates of seeking stool-based screening tests for colorectal cancer among adults aged 45–75 years and found it to be cogent. Specifically, the constructs of participatory dialogue, behavioral confidence, and changes in the social environment were highly predictive of seeking stool-based CRC screening tests. These constructs need to be epitomized in the next phase of subsequent research endeavors in this direction.

## Figures and Tables

**Table 1:** Comparison of sociodemographics characteristics of the study groups (N = 640).

Variable Name	Categories	Overall Sample	Had Any Form of Stool-Based Colorectal Cancer Screening		*p* Value	95% Confidence Interval
		N = 640	Group 1 Yes (*n* = 251)	Group 2 No (*n* = 389)		
Age in years (M ± SD)	-	58.26 ± 8.96	59.08 ± 8.94	57.80 ± 8.94	0.1	57.56, 58.96
Gender	Male	271 (42.4)	113 (45.0)	158 (40.7)	0.4	38.4, 46.2
	Female	368 (57.6)	138 (55.0)	230 (59.3)		53.5, 61.3
Race	Black	74 (11.6)	28 (17.1)	46 (15.3)	0.6	9.0, 14.0
	White	331 (51.7)	116 (70.7)	215 (71.7)		47.7, 55.6
	AAPI	23 (3.6)	10 (6.1)	13 (4.3)		2.3, 5.3
	Other	36 (5.6)	10 (6.1)	26 (8.7)		3.8, 7.4
Ethnicity	Hispanic	86 (13.4)	32 (15.0)	54 (14.0)	0.7	10.8, 16.0
	Non-Hispanic	513 (80.2)	182 (85.0)	331 (86.0)		77.0, 83.2
Marital status	Married	303 (47.3)	114 (52.1)	189 (49.1)	0.4	43.5, 51.2
	Divorced/Separated	123 (19.2)	39 (17.8)	84 (21.8)		16.1, 22.2
	Widowed	42 (6.5)	19 (8.7)	23 (6.0)		4.6, 8.5
	Single, never married	103 (16.1)	38 (17.4)	65 (16.9)		13.2, 18.9
	Other	33 (5.2)	9 (4.1)	24 (6.2)		3.4, 6.8
Education	Less than a high school	17 (2.6)	5 (2.3)	12 (3.1)	0.8	1.4, 3.9
	High school diploma or GED	154 (24.0)	53 (24.2)	101 (26.2)		20.7, 27.3
	Some college but not degree	161 (25.2)	64 (29.2)	97 (25.1)		21.7, 28.5
	College degree	205 (32.0)	71 (32.4)	134 (34.7)		28.4, 35.6
	Graduate level degree	61 (9.5)	24 (11.0)	37 (9.6)		7.2, 11.8
	Other	7 (1.2)	2 (0.9)	5 (1.3)		0.2, 1.9
Health insurance	Yes	567 (88.5)	213 (97.3)	354 (91.9)	0.009	86.1, 91.0
	No	37 (5.8)	6 (2.7)	31 (8.1)		3.9, 7.6
Region	Rural	176 (27.5)	63 (28.8)	113 (29.3)	0.4	24.0, 30.9
	Urban	152 (23.7)	62 (28.3)	90 (23.3)		20.5, 27.1
	Suburban	277 (43.2)	94 (42.9)	183 (47.4)		39.4, 47.1
Employment status	Employed or self employed	279 (43.5)	97 (44.3)	182 (47.2)	0.8	39.7, 47.4
	Not working (e.g., out of work, homemaker, retired)	260 (40.6)	98 (44.7)	162 (42.0)		36.8, 44.4
	Unable to work	66 (10.3)	24 (11.0)	42 (10.9)		7.9, 12.7
Religion	Christian	429 (67.0)	153 (69.9)	276 (71.5)	0.7	63.3, 70.6
	Non-Christian	176 (27.5)	66 (30.1)	110 (28.5)		24.0, 30.9
Median income	<USD 25,000	143 (22.3)	49 (22.9)	94 (25.5)	0.2	19.1, 25.5
	USD 25,000–USD 50,000	162 (25.3)	61 (28.5)	101 (27.4)		21.9, 28.6
	USD 50,001–USD 75,000	111 (17.3)	42 (19.6)	69 (18.7)		14.4, 20.3
	USD 75,001–USD 100,000	57 (8.9)	24 (11.2)	33 (8.9)		6.7, 11.1
	USD 100,001–USD 125,000	40 (6.2)	14 (6.5)	26 (7.0)		4.4, 8.1
	USD 125,001–USD 150,000	33 (5.2)	17 (7.9)	16 (4.3)		3.4, 6.8
	>USD 150,000	37 (5.7)	7 (3.3)	30 (8.1)		3.9, 7.6

Group 1: Who have had any sort of stool-based colorectal screening tests, including fecal immunochemical test (FIT), guaiac-based fecal occult blood test (gFOBT), or Cologuard or multi-targeted stool DNA test (mt-sDNA) tests; Group 2: Who have not had any sort of stool-based colorectal screening tests, including fecal immunochemical test (FIT), guaiac-based fecal occult blood test (gFOBT), or Cologuard or multi-targeted stool DNA test (mt-sDNA) tests. *p* values less than 0.05 are considered statistically significant; percentage may not add up to 100% due to some missing data.

**Table 2:** History of pre-existing illness and screening among the study groups (N = 640).

Variable Name	Categories	Overall Sample *n* (%)	Had Any Form of Stool-Based Colorectal Cancer Screening		*p* Value
		N = 640	Group 1 Yes (*n* = 251)	Group 2 No (*n* = 389)	
Personal history of colorectal cancer	Yes	16 (2.7)	12 (5.6)	4 (1.1)	0.001
	No	577 (97.3)	204 (94.4)	373 (98.9)	
Family history of colorectal cancer	Yes	87 (15.3)	37 (18.0)	50 (13.8)	0.2
	No	481 (84.7)	169 (82.0)	312 (86.2)	
Personal history of inflammatory bowel disease (ulcerative colitis or Crohn’s disease)	Yes	50 (8.7)	27 (13.0)	23 (6.2)	0.005
	No	528 (91.3)	181 (87.0)	347 (93.8)	
Personal history of confirmed or suspected hereditary colorectal cancer syndrome	Yes	20 (3.7)	14 (7.0)	6 (1.7)	0.001
	No	527 (96.3)	185 (93.0)	342 (98.3)	
Personal history of getting radiation to the abdomen (belly) or pelvic area to treat any prior cancer	Yes	30 (5.0)	20 (9.2)	10 (2.6)	<0.001
	No	565 (95.0)	197 (90.8)	368 (97.4)	
Recommended a colorectal screening by healthcare provider (HCP)	Yes	196 (34.8)	93 (45.4)	103 (28.7)	<0.001
	No	345 (61.2)	110 (53.7)	235 (65.5)	
	Do not have HCP	23 (4.1)	2 (1.0)	21 (5.8)	
Encouraged by a family member to undertake CRC screening	Yes	195 (32.2)	78 (35.5)	117 (30.3)	0.2
	No	411 (67.8)	142 (64.5)	269 (69.7)	
Have had a recent visit to a primary healthcare provider	Yes	403 (68.7)	171 (78.4)	232 (62.9)	<0.001
	No	184 (31.3)	47 (21.6)	137 (37.1)	

Group 1: Who have had any sort of stool-based colorectal screening tests, including fecal immunochemical test (FIT), guaiac-based fecal occult blood test (gFOBT), or Cologuard or multi-targeted stool DNA test (mt-sDNA) tests; Group 2: Who have not had any sort of stool-based colorectal screening tests, including fecal immunochemical test (FIT), guaiac-based fecal occult blood test (gFOBT), or Cologuard or multi-targeted stool DNA test (mt-sDNA) tests. *p* values less than 0.05 are considered statistically significant.

**Table 3:** Mean scores and ranges of MTM constructs of initiation (N = 640).

MTM Construct	Had Any Form of Colorectal Cancer Screening		*p* Value	Mean Difference	95% CI of Mean Difference
	Yes (*n* = 251)	No (*n* = 389)			
Overall Initiation Score	2.75 ± 1.16	2.22 ± 1.36	**<0.001**	0.538	0.331, 0.745
Subscales					
Perceived Advantages	14.19 ± 5.09	13.04 ± 5.47	**0.01**	1.14	0.260, 2.039
Perceived Disadvantages	9.50 ± 4.59	9.57 ± 4.80	0.85	−0.076	−0.864, 0.711
Participatory Dialogue	4.69 ± 6.43	3.46 ± 6.50	**0.03**	1.22	0.149, 2.303
Behavioral Confidence	12.10 ± 5.34	11.11 ± 5.53	**0.03**	0.987	0.757, 1.899
Changes in the Physical Environment	8.45 ± 3.31	7.64 ± 3.70	**0.006**	0.817	0.240, 1.393
Change in the Social Environment	7.51 ± 3.61	6.60 ± 3.87	**0.004**	0.916	0.286, 1.546

*p* values less than 0.05 are considered statistically significant and are bolded in the table; all values are represented as the mean and standard deviation.

**Table 4:** Pearson correlations between MTM constructs used in this study.

Variables	1	2	3	4
1. Participatory Dialogue	1	0.510 ** [0.448, 0.567]	0.447 ** [0.380, 0.508]	0.417 ** [0.349, 0.481]
2. Behavioral Confidence	0.510 ** [0.448, 0.567]	1	0.745 ** [0.707, 0.778]	0.676 ** [0.631, 0.718]
3. Changes in the Physical Environment	0.447 ** [0.380, 0.508]	0.745 ** [0.707, 0.778]	1	0.765 ** [0.729, 0.796]
4. Changes in the Social Environment	0.417 ** [0.349, 0.481]	0.676 ** [0.631, 0.718]	0.765 ** [0.729, 0.796]	1
Cronbach’s Alpha	-	0.913	0.874	0.873

Global Alpha = 0.942; ** *p* < 0.01; “participatory dialogue” was measured through “perceived advantages” and “perceived disadvantages”.

**Table 5:** Hierarchical multiple regression to investigate predictors among those who have had any form of colorectal cancer screening (*n* = 251).

Variables	Model 1		Model 2		Model 3		Model 4		Model 5	
	B	β	B	β	B	β	B	β	B	β
Constant	0.120		1.065 *		0.457		0.482		0.601	
Age	0.011	0.071	0.002	0.013	0.000	−0.003	−0.001	−0.004	−0.004	−0.029
Gender: Male (Ref: Female)	−0.071	−0.025	−0.197	−0.070	−0.101	−0.036	−0.106	−0.038	−0.049	−0.017
Race: White (Ref: Black)	0.026	0.009	−0.063	−0.023	−0.128	−0.046	−0.126	−0.045	−0.104	−0.038
AAPI	−1.023 *	−0.128	−0.702	−0.088	−0.524	−0.065	−0.502	−0.063	−0.398	−0.050
Other	−0.225	−0.042	−0.127	−0.024	−0.240	−0.044	−0.232	−0.043	−0.227	−0.042
Ethnicity: Non-Hispanic (Ref: Hispanic)	0.048	0.012	−0.063	−0.016	−0.053	−0.013	−0.066	−0.016	−0.032	−0.008
Marital Status: Divorced/Separated (Ref: Married)	−0.229	−0.068	−0.219	−0.065	−0.166	−0.049	−0.180	−0.053	−0.068	−0.020
Widowed	−0.099	−0.018	0.014	0.002	−0.060	−0.011	−0.073	−0.013	0.020	0.004
Single, never married	−0.374	−0.100	−0.283	−0.076	−0.254	−0.068	−0.281	−0.075	−0.268	−0.071
Other	−0.193	−0.034	−0.034	−0.006	−0.027	−0.005	−0.090	−0.016	−0.164	−0.029
Education Less than a high school (Ref: High school diploma or GED)	−0.270	−0.037	−0.218	−0.030	−0.228	−0.031	−0.254	−0.035	−0.155	−0.021
Some college but not degree	0.211	0.063	0.044	0.013	−0.177	−0.053	−0.175	−0.053	−0.091	−0.027
College degree	0.156	0.054	−0.120	−0.042	−0.299 *	−0.104	−0.322 *	−0.112	−0.283	−0.098
Graduate level degree	−0.155	−0.034	−0.193	−0.042	−0.363	−0.079	−0.369	−0.081	−0.352	−0.077
Other	−0.247	−0.020	−0.445	−0.036	−0.615	−0.049	−0.656	−0.052	−0.710	−0.057
Health insurance: Yes (Ref: No)	1.102 **	0.224	0.660 *	0.134	0.415 *	0.084	0.363	0.074	0.260	0.053
Region: Urban (Ref: Rural)	0.296	0.090	0.224	0.068	0.293	0.089	0.306 *	0.093	0.358 *	0.109
Suburban	0.428 *	0.154	0.262	0.094	0.182	0.066	0.172	0.062	0.190	0.069
Employment status: Not Working (Ref: Employed or self-employed)	0.127	0.045	−0.022	−0.008	−0.045	−0.016	−0.067	−0.024	−0.030	−0.011
Unable to work	−0.101	−0.021	−0.201	−0.041	−0.234	−0.048	−0.247	−0.051	−0.177	−0.036
Religion: Christian (Ref: Non-Christian)	0.137	0.044	0.196	0.063	0.240 *	0.077	0.218	0.070	0.162	0.052
Income: USD 25,000–USD 50,000 (Ref: <USD 25,000)	−0.127	−0.041	−0.039	−0.012	−0.085	−0.027	−0.099	−0.032	−0.130	−0.042
USD 50,001–USD 75,000	0.003	0.001	−0.003	−0.001	−0.140	−0.039	−0.167	−0.046	−0.241	−0.067
USD 75,001–USD 100,000	0.766 *	0.145	0.459	0.087	0.153	0.029	0.111	0.021	0.056	0.011
USD 100,001–USD 125,000	0.229	0.042	0.340	0.062	0.211	0.038	0.203	0.037	0.096	0.017
USD 125,001–USD 150,000	0.471	0.067	0.545	0.077	0.336	0.048	0.280	0.040	0.285	0.040
>USD 150,000	0.275	0.054	0.222	0.043	−0.022	−0.004	−0.050	−0.010	−0.098	−0.019
Personal history of colorectal cancer: Yes (Ref: No)	0.684	0.047	1.003	0.069	1.208 *	0.084	1.181 *	0.082	0.993	0.069
Family history of colorectal cancer: Yes (Ref: No)	0.195	0.046	0.003	0.001	−0.132	−0.031	−0.147	−0.035	−0.120	−0.028
Personal history of inflammatory bowel disease (ulcerative colitis or Crohn’s disease): Yes (Ref: No)	0.194	0.033	−0.069	−0.012	−0.038	−0.006	−0.032	−0.006	−0.057	−0.010
Participatory dialogue	-	-	0.125 **	0.590	0.067 **	0.315	0.066 **	0.310	0.061 **	0.290
Behavioral confidence	-	-	-	-	0.125 **	0.506	0.109 **	0.438	0.096 **	0.388
Changes in the physical environment	-	-	-	-	-	-	0.039	0.102	−0.014	−0.036
Changes in the Social Environment	-	-	-	-	-	-	-	-	0.099 **	0.274
R^2^	0.189	-	0.485	-	0.621	-	0.625	-	0.651	-
F	2.257 **	-	8.826 **	-	14.798 **	-	14.538 **	-	15.730 **	-
ΔR^2^	0.189	-	0.297	-	0.136	-	0.004	-	0.026	-
ΔF	2.257 **	-	167.232 **	-	103.352 **	-	2.976	-	21.286 **	-

* *p*-value < 0.05; ** *p*-value < 0.001; adjusted R^2^ of Model 5 = 0.609.

**Table 6:** Hierarchical multiple regression to investigate predictors among those who have not had any form of colorectal cancer screening (*n* = 389).

Variables	Model 1		Model 2		Model 3		Model 4		Model 5	
	B	β	B	β	B	β	B	β	B	β
Constant	1.790		2.048 *		0.874		1.076		1.171	
Age	0.005	0.038	−0.003	−0.025	0.007	0.053	0.002	0.016	0.000	0.003
Gender: Male (Ref: Female)	−0.012	−0.005	0.143	0.061	0.101	0.043	0.150	0.064	0.190	0.081
Race: White (Ref: Black)	0.063	0.027	0.043	0.018	−0.054	−0.023	−0.062	−0.026	−0.034	−0.014
AAPI	0.360	0.067	0.410	0.076	0.823 *	0.153	0.647	0.120	0.726 *	0.135
Other	0.332	0.065	0.297	0.058	0.140	0.027	0.134	0.026	0.125	0.024
Ethnicity: Non-Hispanic (Ref: Hispanic)	−0.191	−0.060	−0.239	−0.075	−0.304	−0.095	−0.306	−0.096	−0.302	−0.095
Marital Status: Divorced/Separated (Ref: Married)	−0.199	−0.064	−0.012	−0.004	−0.077	−0.025	−0.071	−0.023	−0.069	−0.022
Widowed	0.255	0.062	0.244	0.059	0.085	0.021	0.101	0.024	0.232	0.056
Single, never married	−0.391	−0.129	−0.313	−0.103	−0.220	−0.072	−0.184	−0.061	−0.160	−0.053
Other	−0.504	−0.077	−0.269	−0.041	−0.441	−0.067	−0.306	−0.047	−0.286	−0.044
Education Less than a high school (Ref: High school diploma or GED)	−0.274	−0.034	0.055	0.007	0.344	0.043	0.193	0.024	0.104	0.013
Some college but not degree	−0.170	−0.065	−0.158	−0.060	−0.042	−0.016	−0.140	−0.054	−0.094	−0.036
College degree	0.048	0.019	−0.111	−0.043	−0.140	−0.055	−0.137	−0.054	0.030	0.012
Graduate level degree	0.221	0.063	0.163	0.046	0.205	0.058	0.081	0.023	0.131	0.037
Other	−2.249 *	−0.201	−1.666 *	−0.149	−1.437 *	−0.128	−1.420 *	−0.127	−1.166	−0.104
Health insurance: Yes (Ref: No)	0.985	0.150	0.948 *	0.145	0.568	0.087	0.382	0.058	0.273	0.042
Region: Urban (Ref: Rural)	−0.154	−0.058	−0.216	−0.082	−0.194	−0.074	−0.227	−0.086	−0.261	−0.099
Suburban	−0.035	−0.015	−0.057	−0.024	−0.057	−0.024	−0.057	−0.024	−0.040	−0.017
Employment status: Not Working (Ref: Employed or self-employed)	−0.010	−0.004	−0.208	−0.088	−0.318	−0.135	−0.300	−0.127	−0.282	−0.119
Unable to work	0.268	0.073	0.034	0.009	0.041	0.011	−0.001	0.000	−0.052	−0.014
Religion: Christian (Ref: Non-Christian)	−0.218	−0.086	−0.170	−0.067	−0.047	−0.019	−0.081	−0.032	−0.012	−0.005
Income: USD 25,000–USD 50,000 (Ref: <USD 25,000)	−0.116	−0.045	−0.220	−0.085	−0.166	−0.064	−0.191	−0.074	−0.318	−0.123
USD 50,001–USD 75,000	0.368	0.118	0.141	0.045	0.075	0.024	0.019	0.006	−0.171	−0.055
USD 75,001–USD 100,000	0.157	0.042	0.205	0.055	0.143	0.038	0.044	0.012	−0.088	−0.024
USD 100,001–USD 125,000	0.374	0.079	0.198	0.042	0.157	0.033	0.047	0.010	−0.162	−0.034
USD 125,001–USD 150,000	0.860	0.202	0.581	0.137	0.470	0.111	0.406	0.096	0.254	0.060
>USD 150,000	0.504	0.083	0.359	0.059	0.372	0.061	0.300	0.049	0.089	0.015
Personal history of colorectal cancer: Yes (Ref: No)	−0.093	−0.017	0.028	0.005	−0.202	−0.037	−0.083	−0.015	0.039	0.007
Family history of colorectal cancer: Yes (Ref: No)	0.039	0.012	0.065	0.021	−0.106	−0.034	−0.055	−0.017	−0.030	−0.009
Personal history of inflammatory bowel disease (ulcerative colitis or Crohn’s disease): Yes (Ref: No)	−0.255	−0.074	−0.156	−0.045	−0.099	−0.029	−0.105	−0.030	−0.100	−0.029
Participatory dialogue	-	-	0.084 **	0.457	0.052 **	0.283	0.045 *	0.244	0.042 *	0.229
Behavioral confidence	-	-	-	-	0.099 **	0.446	0.063 *	0.283	0.043 *	0.193
Changes in the physical environment	-	-	-	-	-	-	0.096 *	0.263	0.033	0.092
Changes in the Social Environment	-	-	-	-	-	-	-	-	0.108 *	0.331
R^2^	0.208	-	0.373	-	0.516	-	0.545	-	0.579	-
F	1.313	-	2.856 **	-	4.934 **	-	5.334 **	-	5.916 **	-
ΔR^2^	0.208	-	0.165	-	0.143	-	0.029	-	0.035	-
ΔF	1.313	-	39.128 **	-	43.879 **	-	9.287 *	-	11.977 *	-

* *p*-value < 0.05; ** *p*-value < 0.001; Adjusted R^2^ for Model 5 = 0.481.

**Table 7:** Possible script for suggesting Cologuard to an eligible patient.

MTM Construct	Crib Sheet Lines
Advantages	Cologuard is a non-invasive, home-based stool test for the early detection of polyps and colon cancer for people 45 years of age and older.
Behavioral confidence	It is very easy to use and comes with all detailed instructions.
Changes in the social environment	Should you decide to use it, our nurse will be able to explain all preliminary details to you.

## Data Availability

The data presented in this study are available on request from the corresponding author. The data are not publicly available due to ethical reasons.

## Supplementary Materials

The following supporting information can be downloaded at: https://www.mdpi.com/article/10.3390/ijerph20166553/s1.
