# Supplementary material for: Using the Multi-Theory Model (MTM) of Health Behavior Change to Explain the Seeking of Stool-Based Tests for Colorectal Cancer Screening
Source: Int J Environ Res Public Health. 2023 Aug 10;20(16):6553. doi: 10.3390/ijerph20166553 (PMC10454677; doi:10.3390/ijerph20166553)
Supplement: Supplementary file 1 [file ijerph-20-06553-s001.zip › ijerph-2479136-supplementary.pdf]

# MEASURING CHANGE IN COLORECTAL CANCER SCREENING

Directions: American Cancer Society recommends a variety of screening tests for colorectal cancer screening in people in the age group 45 to 75 years. The stool-based tests are:

- Highly sensitive fecal immunochemical test (FIT) every year
- Highly sensitive guaiac-based fecal occult blood test (gFOBT) every year
- Cologuard or Multi-targeted stool DNA test (mt-sDNA) every 3 years

The visual (structural) exams of the colon and rectum-based tests are:

- Colonoscopy every 10 years
- CT colonography (virtual colonoscopy) every 5 years
- Flexible sigmoidoscopy (FSIG) every 5 years.

The following questions are about these tests. You may or may not have taken any one or more of these tests but provide a response to the best of your abilities.

1. Do you belong to the age group 45-75 years? Yes
   No

   *If your response is "no," please stop taking the survey.*

…………………………………………………………………………………………………

2. Have you had a fecal immunochemical test (FIT) in the past year? Yes
   No

…………………………………………………………………………………………………

3. If you had a fecal immunochemical test (FIT) in the past year, was it normal?
   Yes
   No, if not, did you follow up with a doctor: Yes No
   Not applicable

…………………………………………………………………………………………………

4. Have you had a guaiac-based fecal occult blood test (gFOBT) in the past year?
   Yes
   No

…………………………………………………………………………………………………

5. If you had a guaiac-based fecal occult blood test (gFOBT) in the past year, was it normal?
   Yes
   No, if not, did you follow up with a doctor: Yes No
   Not applicable

…………………………………………………………………………………………………

6. Have you had a Cologuard (mt-sDNA) test in the past 3 years? Yes
   No

…………………………………………………………………………………………………

7. If you had Cologuard (mt-sDNA) test in the past 3 years, was it normal?
   Yes
   No, if not, did you follow up with a doctor: Yes No
   Not applicable

…………………………………………………………………………………………………

8. Have you had a colonoscopy in the past 10 years? Yes
   No

…………………………………………………………………………………………………

9. If you had a colonoscopy in the past 10 years, was it normal?
   Yes
   No, if not, did you follow up with a doctor: Yes No
   Not applicable

…………………………………………………………………………………………………

10. Have you had a CT colonography (virtual colonoscopy) in the past 5 years?
    Yes
    No

…………………………………………………………………………………………………

11. If you had a CT colonography (virtual colonoscopy) in the past 5 years, was it normal?
    Yes
    No, if not, did you follow up with a doctor: Yes No
    Not applicable

…………………………………………………………………………………………………

12. Have you had a Flexible sigmoidoscopy (FSIG) in the past 5 years?
    Yes
    No

…………………………………………………………………………………………………

13. If you had a Flexible sigmoidoscopy (FSIG) in the past 5 years, was it normal?
    Yes
    No
    Not applicable

…………………………………………………………………………………………………

14. Are you hesitant to undergo any form of colorectal cancer screening?
    Yes
    No
    Don't know

…………………………………………………………………………………………………

15. Have you had a recent visit within the past three months to a primary health care provider (doctor)? Yes
    No
    Not applicable

…………………………………………………………………………………………………

16. Have you been recommended a colorectal cancer screening by your health care provider (doctor)? Yes
    No

…………………………………………………………………………………………………

17. Have you been encouraged by a family member to get a colorectal cancer screening? Yes
No

…………………………………………………………………………………………………………

18. What is your gender? Male
Female
Other, please specify ________________
Prefer not to answer

…………………………………………………………………………………………………………

19. How old are you today? _______ years

…………………………………………………………………………………………………………

20. How long have you lived in the United States? _______

…………………………………………………………………………………………………………

21. With which racial/ ethnic group do you associate yourself (you may mark more than one)?
Alaska Native
American Indian
Asian
Black or African American
Caucasian American or White
Hispanic, Latina, or Latinx
Native Hawaiians or Pacific Islanders
Other, please specify ______________________
Prefer not to answer

…………………………………………………………………………………………………………

22. What is your employment status?
Employed for wages
Self-employed
Out of work
A homemaker
Retired
Unable to work
Other, please specify _____________

…………………………………………………………………………………………………………

23. What is your religion? Christianity
Islam
Buddhism
Judaism
Atheist
Hinduism
Other, please specify ___________________

…………………………………………………………………………………………………………

24. Where do you live? Rural
Urban

Suburban

…………………………………………………………………………………………………………

25. What is your 5-digit zip code? ________________

…………………………………………………………………………………………………………

26. What is the highest level of education you have completed?

Less than a high school diploma
High school graduate (or equivalent including GED)
Some college but no degree
College Degree (Associate or Bachelor's)
Graduate Degree
Other ___________

…………………………………………………………………………………………………………

27. Do you have health insurance? Yes, If so, please choose Private Medicare Medicaid Tricare Other, please specify ________
No

…………………………………………………………………………………………………………

28. What is your yearly household income?

Less than $25,000
$25,000 - $50,000
$50,001 - $75,000
$75,001 - $100,000
$100,001 - $125,000
$125,001 - $150,000
Greater than $150,001
Prefer not to answer

…………………………………………………………………………………………………………

29. What is your marital status?

Married
Divorced
Widowed
Separated
Never married
In a civil union or registered domestic partnership
A member of an unmarried couple

…………………………………………………………………………………………………………

30. Do you suffer from any of the following chronic diseases? (Check all that apply) Heart disease

Any type of cancer
Chronic Obstructive Lung Disease (COPD)
Depression
Chronic kidney disease
Diabetes
Arthritis
Other________

…………………………………………………………………………………………………………

31. Do you have a personal history of colorectal cancer?
Yes
No
Don't know

…………………………………………………………………………………………………

32. Do you have a family history of colorectal cancer?
Yes
No
Don't know

…………………………………………………………………………………………………

33. Do you have a personal history of inflammatory bowel disease (ulcerative colitis or Crohn's disease)?
Yes
No
Don't know

…………………………………………………………………………………………………

34. Do you have a personal history of confirmed or suspected hereditary colorectal cancer syndrome, such as familial adenomatous polyposis (FAP) or Lynch syndrome (hereditary non-polyposis colorectal cancer or HNPCC)?
Yes
No
Don't know

…………………………………………………………………………………………………

35. Do you have a personal history of getting radiation to the abdomen (belly) or pelvic area to treat any prior cancer?
Yes
No
Don't know

…………………………………………………………………………………………………

Please provide the response that denotes your best position on the following questions. There are no correct or incorrect responses.

| | Never | Almost Never | Sometimes | Fairly Often | Very Often |
|---|---|---|---|---|---|
| 36. If you get a colorectal cancer screening, you will find out if you have colorectal cancer earlier. | | | | | |
| 37. If you get a colorectal cancer screening, you will increase the chances of a cure. | | | | | |
| 38. If you get a colorectal cancer screening, you will have peace of mind. | | | | | |

39. If you get a colorectal cancer screening, your worries will be reduced.

40. If you get a colorectal cancer screening, you will increase your chances of living longer.

| | Never | Almost Never | Sometimes | Fairly Often | Very Often |
|---|---|---|---|---|---|

41. When you need to get a colorectal cancer screening, it may cause anxiety.

42. When you need to get a colorectal cancer screening, it may cause physical discomfort/pain.

43. When you need to get a colorectal cancer screening, you may feel an invasion of your modesty.

44. When you need to get a colorectal cancer screening, it may cause embarrassment.

45. When you need to get a colorectal cancer screening, you may get a misdiagnosis.

| | Not At All Sure | Slightly Sure | Moderately Sure | Very Sure | Completely Sure |
|---|---|---|---|---|---|

46. How sure are you that you can get a colorectal cancer screening when it is due?

47. How sure are you that you can get a colorectal cancer screening despite physical discomfort/pain?

48. How sure are you that you can get a colorectal cancer screening despite its cost?

49. How sure are you that you can get a colorectal cancer screening despite anxiety?

50. How sure are you that you can get a colorectal cancer screening despite being pressed for time?

| | Not At All Sure | Slightly Sure | Moderately Sure | Very Sure | Completely Sure |
|---|---|---|---|---|---|

51. How sure are you that you can find a location to get a colorectal cancer screening?

52. How sure are you that you can travel to a place to get a colorectal cancer screening?

53. How sure are you that you can afford to get a colorectal cancer screening?

| | Not At All Sure | Slightly Sure | Moderately Sure | Very Sure | Completely Sure |
|---|---|---|---|---|---|

54. How sure are you that you can get help from a family member to undergo colorectal cancer screening?

…………………………………………………………………………………………………………

55. How sure are you that you can get help from a friend to undergo colorectal cancer screening?

…………………………………………………………………………………………………………

56. How sure are you that you can get help from a health care professional to undergo colorectal screening?

| | Not At All Likely | Somewhat Likely | Moderately Likely | Very Likely | Completely Likely |
|---|---|---|---|---|---|

57. How likely is it that you will get a colorectal cancer screening when it is due?

…………………………………………………………………………………………………………

*Thank you for your time!*

## SCORING

**Construct of advantages:** Scale: Never (0), Almost never (1), Sometimes (2), Fairly often (3), Very often (4). The summative score of Items 36-40. Possible range: 0- 20 units. A high score is associated with the likelihood of initiation of behavior change.

**Construct of disadvantages:** Scale: Never (0), Almost never (1), Sometimes (2), Fairly often (3), Very often (4). The summative score of Items 41-45. Possible range: 0- 20 units. A low score is associated with the likelihood of initiation of behavior change.

Subtract the disadvantages score from the advantages score to calculate the **participatory dialogue** construct score. Possible range: -20 to +20 units. A positive score will be indicative of behavior change.

**Construct of behavioral confidence:** Scale: Not at all sure (0), slightly sure (1), moderately sure (2), very sure (3), completely sure (4). The summative score of Items 46-50. Possible range 0-20 units. A high score is associated with the likelihood of initiation of behavior change.

**Construct of changes in the physical environment:** Scale: Not at all sure (0), slightly sure (1), moderately sure (2), very sure (3), completely sure (4). The summative score of Items 51-53. Possible range 0-12 units. A high score is associated with the likelihood of initiation of behavior change.

**Construct of changes in the social environment:** Scale: Not at all sure (0), slightly sure (1), moderately sure (2), very sure (3), completely sure (4). The summative score of Items 54-56. Possible range 0-12 units. A high score is associated with the likelihood of initiation of behavior change.

For **modeling initiation,** the dependent variable can be Item 57: not at all likely (0), somewhat likely (1), moderately likely (2), very likely (3), and completely likely (4), and multiple regression can be used.

In addition, logistic regression can be used for each type of screening procedure undertaken in the stipulated time period as the dependent variable to identify socio-demographic and MTM predictors for each.

**Flesch Reading Ease: 62.4**
**Flesch-Kincaid Grade Level: 7.7**

© Manoj Sharma
